# Endoscopy-assisted purely total outer wall excision for pediatric Sylvian arachnoid cysts

**DOI:** 10.1186/s41016-023-00330-7

**Published:** 2023-07-13

**Authors:** Mingxing Wu, Fei Di, Mingle Ma, Jiye Li, Yanbin Li, Bingke Zhang

**Affiliations:** Department of Neurosurgery, The Affiliated Children’s Hospital, Capital Institute of Pediatrics, Beijing, China

**Keywords:** Arachnoid cysts, Endoscopy, Treatment

## Abstract

**Background:**

To present a novel endoscopy-assisted surgical strategy of Sylvian arachnoid cysts (ACs).

**Case presentation:**

Endoscopy-assisted surgery was performed on 9 children (May 2019–December 2021). All patients were evaluated with CT and/or MRI and had regular follow-up examinations. The procedure consisted of performing a small temporal craniotomy (2 cm) behind the hairline. After dural opening, the surgery was performed with the assistance of a rigid 30-degree transcranial endoscope, self-irrigating bipolar forceps, and other standard endoscopic instruments. Steps included total excision of the AC outer wall and dissection of arachnoid adhesion around the cystic edge to communicate the residual cyst cavity with subdural space. Compared with the microscopical procedure, a 30-degree transcranial endoscope provides a wider view, especially for the lateral part exposure of the outer wall.

The average age of the patients was 27.7 months (range 13–44 months). The Sylvian AC was in the right hemisphere in three patients and six in the left, respectively. 1 patient suffered transient postoperative epilepsy. There was no mortality or additional postoperative neurological deficit in this series. All of the patients achieved significant clinical improvement after surgery. Radiological examination after the operation showed a significant reduction in all cases (100%, 9/9) and disappearance in one case (11.1%, 1/9). Postoperative subdural fluid collection occurred in six cases and completely resolved spontaneously in 9 months.

**Conclusion:**

The study demonstrated the minimally invasive, safety, and effectivity of the endoscopy-assisted purely total outer wall excision.

## Background

Arachnoid cysts (ACs) are known as congenital lesions, which are often located in relation to an arachnoid cistern. Sylvian cysts are the most common of the ACs, accounting for approximately one-half of the patients [1, 2]. Most authors recommend not treating Sylvian ACs that do not cause mass effects or symptoms, regardless of their size. Three surgical treatment options exist for Sylvian ACs, including craniotomy excising cyst wall and fenestrating it into basal cisterns simultaneously, endoscopic cyst fenestration through a burr hole, or shunting of cyst. The optimal surgical technique remains controversial [3–6]. Shunting of cyst is an effective surgical treatment; however, shunt revision for various reasons rates of 39% [1]. Shunt dependency syndrome is a rare but serious complication of shunting an arachnoid cyst [1, 7]. Endoscopic cyst fenestration is minimally invasive but had earlier complications and a shorter event-free survival than shunting and microsurgery [3]. Here, this article describes a novel endoscopic surgical strategy performed on 9 cases: total excision of the arachnoid outer wall and circumferential dissection of arachnoid adhesion if existed around the cystic edge to communicate the residual cyst cavity with subdural space.

## Case presentation

**Fig. 1:**
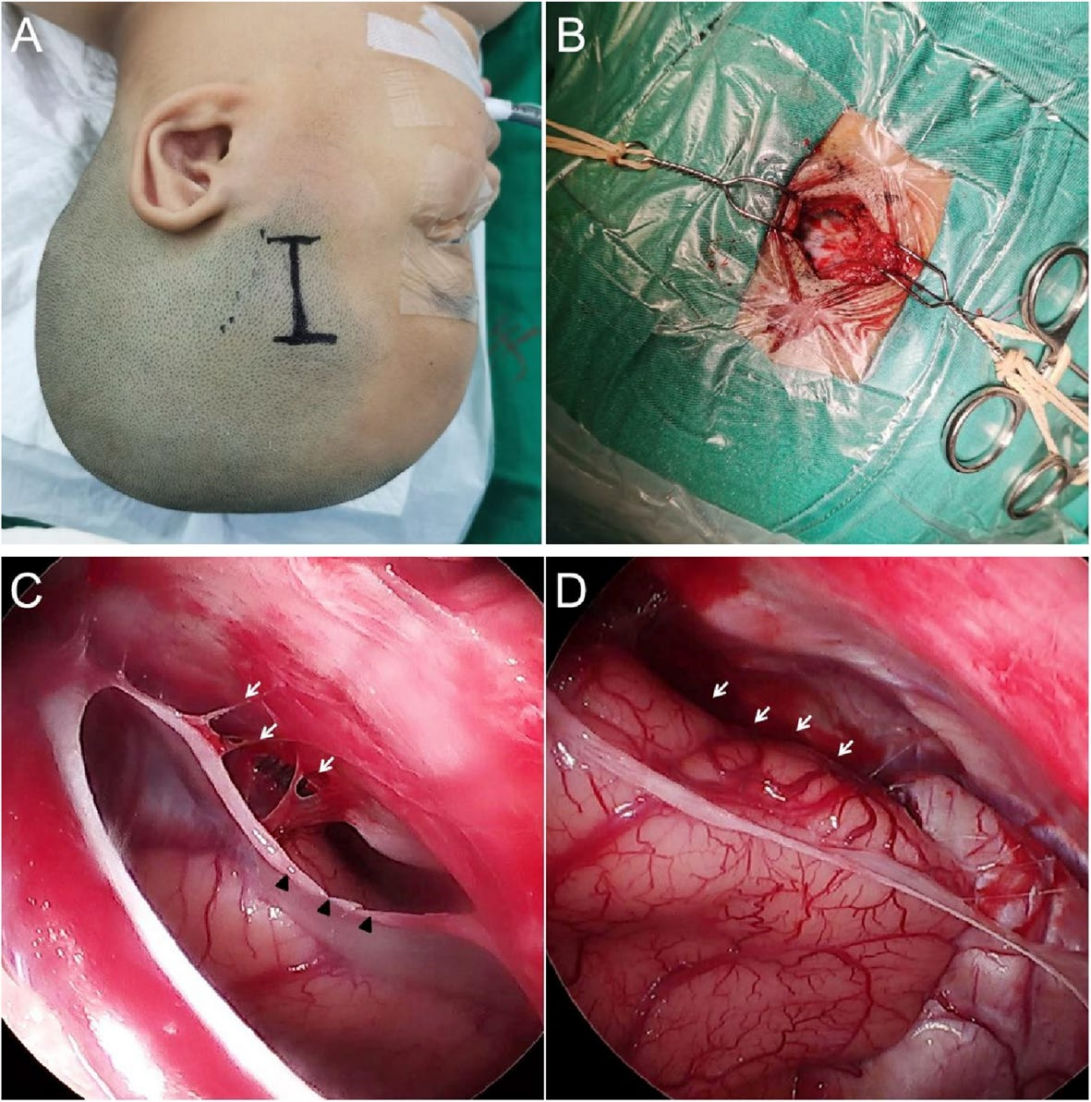
Surgical technique. **A** A linear incision about 3 cm over the temporal muscle was made behind the hairline. **B** A temporal craniotomy 2 cm in diameter was performed. **C** The endoscope was introduced to the edge of cyst (arrowheads) and the transition zone where the arachnoid adhesion usually existed with arachnoid trabecula (arrows).**D** The arachnoid adhesion has been dissected. The cystic cavity was open to broad subdural space(arrows)

We performed surgical treatment for symptomatic Sylvian ACs and asymptomatic cysts with prominent mass effect or size progression. All patients underwent preoperative MR imaging and CT scan. A linear incision about 3 cm over the temporal muscle was made behind the hairline(Fig. 1A). A temporal craniotomy 2 cm in diameter was performed (Fig. 1B). In the temporal bulging case, a longer linear incision over the bulging and a craniotomy including the bulging bone top usually 3 cm in diameter was performed in order to do skull plasty. The dura mater was opened. A rigid 30-degree transcranial neuroendoscope (Aesculap, Germany) was used in the surgery. The AC outer wall was totally removed to the cystic edge which transits to the cerebral surface assisted by an endoscope. Superficial Sylvian vein often courses in the midst of the wall, which should be identified and protected carefully. The endoscope was introduced to the edge of cyst and the transition zone where the arachnoid adhesion usually existed with arachnoid trabecula (Fig. 1C). Usually, the adhesion is tighter in the cases presenting with bulging bone than others. The arachnoid adhesion was circumferentially dissected as much as possible in order to communicate the residual cyst cavity with subdural space and untether the involved frontal and temporal lobe (Fig. 1D). It is a crucial procedure to dissect the arachnoid adhesion around the edge of the cyst. The procedure not only opens a pathway for CSF in the cystic cavity to broad and lower resistant subdural space but also untethers the involved lobes. Superficial draining veins often exist along the edge, which should be identified and protected carefully. Compared with conventional microscopy procedure, 30-degree transcranial endoscope provides a wider view, especially for the lateral part exposure of the outer wall covered by the calvaruium.The endoscopic technique provides a multiangled, close-up view of the transition zone from the pia over the cortex to the cystic outer wall, which is invisible under the microscopy via the small bone window. We did not perform any fenestration at the inner wall of the cysts into the basal cisterns. The dura was closed with interrupted sutures, which was followed by the replacement of the bone and multilayer closure of the temporal muscle, fascia, subcutaneous tissue, and skin. In the temporal bulging case, the bone flap underwent plasty to a flat form before replacement. The patients were encouraged lateral sleeping posture with treated side below 1 month after the operation which promoted restoration of the involved brain tissue.

Nine patients were treated with this surgical strategy between May 2019 and December 2021. Their average age was 5.2 years (range 2–13 years). The Sylvian ACs were in the right hemisphere in three patients and left in six, respectively. One case was type I, 3 cases were type II, and 5 cases were type III according to the Galassi classification [8]. Patient characteristics and clinical findings are presented in Table 1. The main preoperative symptoms were skull deformity, headache, mass effect, and progression of AC size. One patient suffered headaches and papilledema after rupture(case 8). All patients underwent surgery successfully without large hemorrhages. Superficial Sylvian was protected well in every case. There was no operative mortality, postoperative hematoma, and postoperative infection. One patient suffered transient postoperative epilepsy without recurrence after short-term antiepileptic drug.

**Table 1:** Patients underwent the novel endoscopic surgical strategy

Patients	Age (years)	Gender	Clinical presentation	Galassi type	Follow-up (months)	Resolution of symptoms	Reduction of cyst size
1	4	M	Progression of AC size and mass effect	II	44	Cure and cure	Near-total disappearance
2	2	M	Headache and skull deformity	III	43	Cure and SI	Total disappearance
3	5	M	Mass effect	III	31	Cure	Near-total disappearance
4	3	M	Skull deformity	III	31	SI	Near-total disappearance
5	5	M	Skull deformity	II	29	SI	Most disappearance
6	4	F	Mass effect	III	21	Cure	Near-total disappearance
7	7	M	Dizziness	III	19	Cure	Most disappearance
8	4	M	Headache and papilledema (rupture)	I	18	Cure	Near-total disappearance
9	13	M	Headache and mass effect	II	13	SI and cure	Most disappearance

*SI*, Significant improvement

Near-total disappearance: volume reduction ≥ 90%

Most disappearance: 90% > volume reduction ≥ 60%

**Fig. 2:**
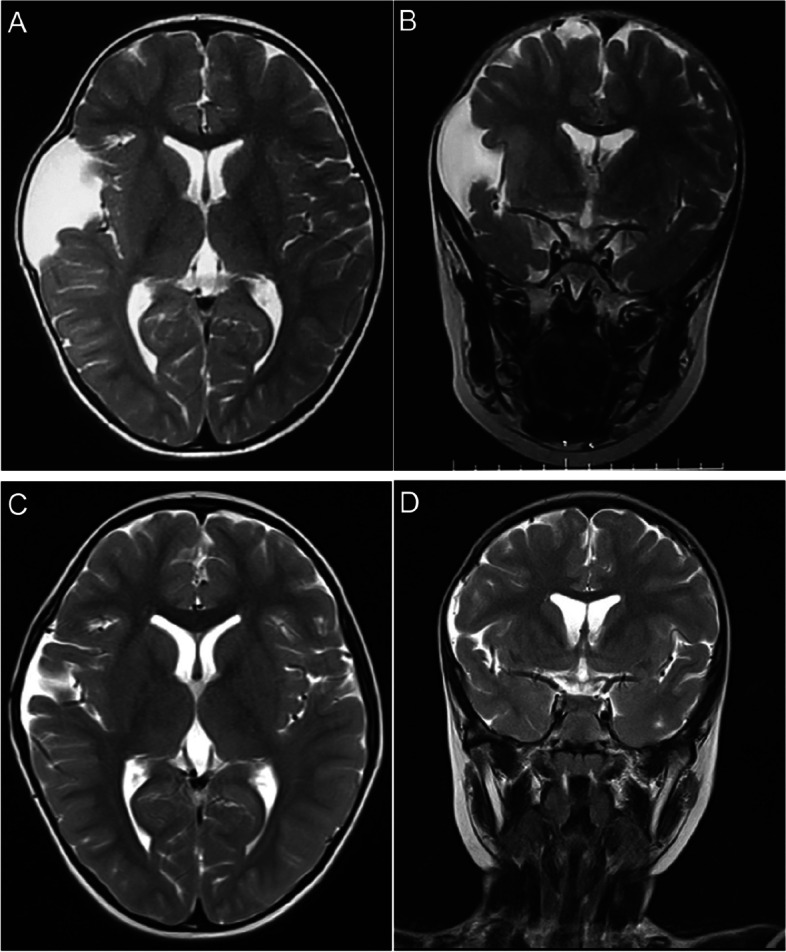
A 2-year-old boy presented with headache and skull deformity. **A**, **B** Preoperative axial and coronal T2-weighted magnetic resonance image of the head showing Sylvian AC and bone bulging on the right. **C**, **D** Axial and coronal T2-weighted MR image showing total disappearance of the cyst on 24 months follow-up MR imaging

**Fig. 3:**
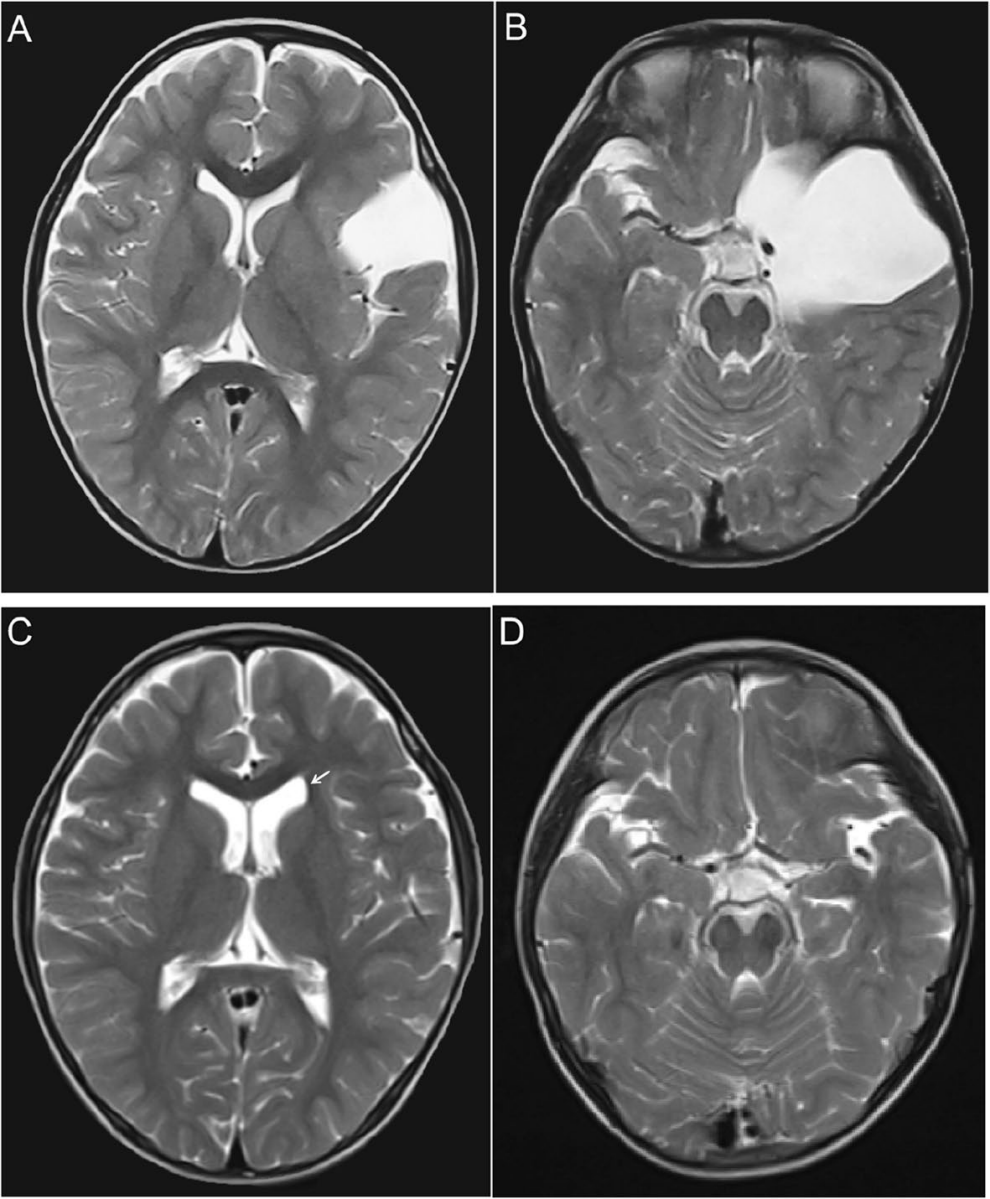
A 4-year-old boy presented with progression of AC size. **A**, **B** Preoperative axial T2-weighted Magnetic resonance image of the head showing Sylvian AC on the left and bilateral symmetrical ventricles.**C**, **D** The same slice of axial T2-weighted MR image showing the disappearance of the cyst and slightly dilated ipsilateral ventricle (arrow) on 12 months follow-up MR imaging

Patients were followed up for an average of 27.7 months (range 13–44 months). All of the patients achieved significant clinical improvement after surgery. Significant improvement of skull deformity occurred in all 3 patients (100%) presenting with temporal bulging. Headache disappeared in 2 patients out of 3 (66.7%) and greatly improved in 1 patient (33.3%). Postoperative MR imaging showed the disappearance of mass effect in all 4 patients (100%). Radiological examination after the operation showed a significant reduction in all cases (100%, 9/9) and total disappearance in 1 case (Fig. 2A–D), near-total disappearance (volume reduction ≥ 90%) in 5 cases, and most disappearance (90% > volume reduction ≥ 60%) in 3 cases, respectively. Postoperative subdural fluid collection occurred in 6 cases without mass effect which didn’t require surgical intervention and completely resolved spontaneously in 9 months. The follow-up images showed an interesting phenomenon that dilated the ipsilateral ventricle in three cases (Fig. 3A–D).

Typical symptoms of Sylvian ACs are seizures, headache, hemiparesis, and skull deformity. Most of the ACs without mass effect or symptoms only need to be followed without treatment. Operative management is indicated for symptomatic, mass effective, or progressive Sylvian ACs [9–12]. Neuroendoscopic management is emerging as a promising alternative procedure that avoids the more invasiveness of traditional open craniotomy and the complications caused by shunting.

Open craniotomy is a traditional surgical treatment. The surgical procedure varies with different authors. E. Galassi et.al. supposed that the extensive resection of the secreting capsular wall and the establishment of a wide communication with the subarachnoid pathways can adequately normalize the fluid circulation. So the procedure includes excision of the removable arachnoid linings and deep perforation of the inner membrane into the basal cisterns in order to enlarge or create a free communication with the CSF pathways [9].Whereas Atsushi Okano et al. only resected the inner membrane and dissected arachnoid membrane including the Liliequist’s membrane around the arteries and nerves [13]. Most of the authors suggested that microsurgical or endoscopic fenestration to create communications between the cyst cavity and basal cisterns was the key [11, 12, 14–16]. An avascular portion of the inner cyst wall is selected as the target area for fenestration, between the optic nerve and the carotid artery, or between the carotid artery and the oculomotor nerve, or under the oculomotor nerve. Pure outer membrane resection without fenestration is rarely reported [17]. Joon-Ki Kang et.al. performed excision of the cyst membrane alone without fenestration in 2 patients but none of them was successful [18]. Morphological and enzyme ultracytochemical evidence was presented to support the contention that the walls of AC secrete fluid [19]. Therefore total excision of the outer walls was the first step in our surgical strategy which would remarkably reduce fluid secreting.

**Fig. 4:**
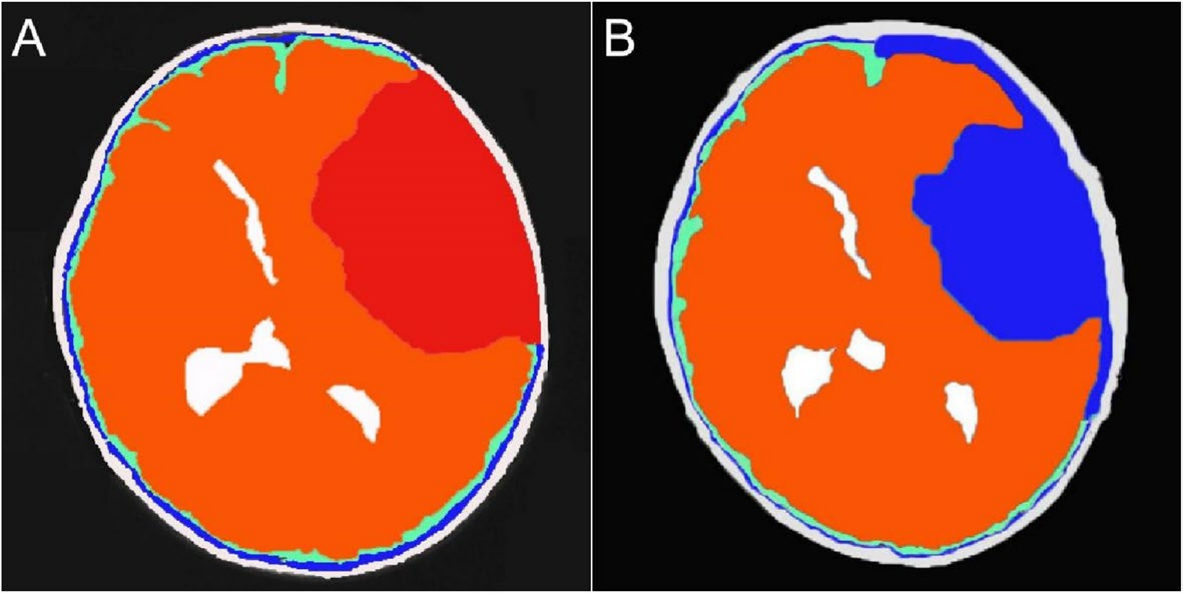
Change of cystic pressure before and after operation. **A** The preoperative pressure from low to high is in the subdural space (blue area), parenchyma (orange area), and cyst (red area), respectively. Red-high pressure area, orange-medium pressure area, blue-low pressure area. **B** After communicating the cyst cavity with broad subdural space, the residual cyst cavity transformed to a low-pressure area equal to subdural space (blue area)

The subdural space pressure is lower than the intracystic and intraparenchymal pressure (Fig. 4A). Circumferential dissection of arachnoid adhesion communicates the residual cyst cavity with subdural space. The pressure gradient facilitates the movement originating from the brain pulse that the CSF in the cavity rushes to the subdural space, which is followed by shifting of the adjacent compressed cerebral tissue(Fig. 4B). Compared with cases fenestrated in the inner cyst wall to the basal cisterns, cystic cavity may be reduced easily in our cases opened to lower resistant broad subdural space. The interesting phenomenon of postoperative dilation of the ipsilateral ventricle in large cases demonstrates the pressure gradient between the parenchyma and cyst cavity which is equal to the subdural space. Furthermore, dissection of arachnoid adhesion untethers the involved brain tissue and weakens the resistance against restoration.

Radiologically,71.9–91.2% of patients demonstrated decreases in cyst size in endoscopically or microsurgically fenestrated series [11, 12, 14–16]. Lower percentage of patients with grade III cysts exhibited evidence of decreases in cyst size than grade I and II during long-term monitoring [11, 16]. However, only a small percentage of the cysts completely disappeared postoperatively, especially rare in the grade III cysts [11, 14, 15]. Michael L. Levy et al. reported seventeen percent of grade III cysts demonstrated complete resolution, but only with follow-up monitoring of more than 5 years [15]. In our series, postoperative MR imaging showed a significant reduction in all cases in the early period.

The rate of clinical improvement is 87.5–92.5% higher than radiological in most of the reports [14, 15]. The symptoms most likely to improve were skull deformity, headache, hemiparesis, and abducens nerve palsies. Cognitive disorders were rarely improved [11, 14–16]. We achieved both clinically and radiologically excellent outcomes for each patient in our series. However, it has a small number of patients and needs long-term follow-up.

The main surgical complication of fenestration was subdural hygroma or hemotoma. Spacca B, et al. reported 5 patients in 40 developed subdural hygroma of whom four required subdural-peritoneal shunting [15]. El-Ghandour NM reported ipsilateral subdural hygroma occurred in 2 cases of 32 (6.3%), which was small in size, asymptomatic, and resolved spontaneously within a few weeks [14]. In the study of Couvreur T et, al. that included 34 cases of Sylvian ACs treated using endoscopy with a subdural hygroma occurred in 3 patients (8.8%) within 3 weeks after surgery, none of them being symptomatic or in need of surgical treatment [16]. The subdura hygroma usually locates between the outer cyst wall and dura. Godano et al. seamed the outer cyst wall circumferentially to the dural edges to prevent its retraction and subdural hygroma formation [12]. Postoperative subdural fluid collection occurred in six cases which did not require surgical intervention in our series. Collapsing of the untethered lobe and spilling of the CSF to subdural space may be the reasons for a high incidence of postoperative fluid collection, which resolved as a decrease of fluid secreting and restoration of the involved lobe. The complication of transient oculomotor nerve paralysis rates from 3.1 to 11.8% in the fenestration series [11, 14, 16]. Our surgical strategy avoids injury risk of a cranial nerve or internal carotid. The 30-degree scope offered a superior view of all of the cyst outer wall by a small craniotomy.

## Conclusions

In this study, we described a novel endoscopy-assisted surgical strategy for Sylvian ACs which was performed in 9 pediatric patients. The study demonstrated the minimally invasive, safety, and effectiveness of this procedure.

## Data Availability

The datasets used or analyzed during the current study are available from the corresponding author on reasonable request.

## Abbreviations

AC: Arachnoid cyst
CSF: Cerebrospinal fluid
